# Peripheral targets attenuate miniature eye movements during fixation

**DOI:** 10.1038/s41598-023-34066-2

**Published:** 2023-05-07

**Authors:** Scott N. J. Watamaniuk, Jeremy B. Badler, Stephen J. Heinen

**Affiliations:** 1 Wright State University, Dayton, USA; 2 The Smith-Kettlewell Eye Research Institute, San Francisco, USA; 3 University of Marburg, Marburg, Germany

**Keywords:** Human behaviour, Psychology, Neuroscience, Oculomotor system, Sensorimotor processing

## Abstract

Fixating a small dot is a universal technique for stabilizing gaze in vision and eye movement research, and for clinical imaging of normal and diseased retinae. During fixation, microsaccades and drifts occur that presumably benefit vision, yet microsaccades compromise image stability and usurp task attention. Previous work suggested that microsaccades and smooth pursuit catch-up saccades are controlled by similar mechanisms. This, and other previous work showing fewer catch-up saccades during smooth pursuit of peripheral targets suggested that a peripheral target might similarly mitigate microsaccades. Here, human observers fixated one of three stimuli: a small central dot, the center of a peripheral, circular array of small dots, or a central/peripheral stimulus created by combining the two. The microsaccade rate was significantly lower with the peripheral array than with the dot. However, inserting the dot into the array increased the microsaccade rate to single-dot levels. Drift speed also decreased with the peripheral array, both with and without the central dot. Eye position variability was higher with the array than with the composite stimulus. The results suggest that analogous to the foveal pursuit, foveating a stationary target engages the saccadic system likely compromising retinal-image stability. In contrast, fixating a peripheral stimulus improves stability, thereby affording better retinal imaging and releasing attention for experimental tasks.

## Introduction

Laboratory experiments that study human and non-human primate visual systems commonly require participants to maintain fixation. This is generally achieved by foveating a small (< 1° visual arc) target, which is usually a high-contrast spot. Fixation is used to stabilize the eyes so that the retinal location of presented visual stimuli remains constant, and also to precisely assess retinal anatomy and function with imaging equipment in the clinic^1^. Fixation is also employed to minimize the retinal-image motion of experimental stimuli, since when an image’s motion exceeds 3°/s, it blurs^2^. However, the goal of image stabilization using spot fixation is not completely realized as the eyes continuously move, exhibiting a characteristic pattern of miniature eye movements consisting of jerky microsaccades and slow, smooth drifts (for review see^3^).

Microsaccades are the most extensively studied miniature eye movement^3^. It has long been believed that microsaccades benefit vision by preventing retinal-image fading that occurs when an image is completely stabilized on the retina^4–6^. However, there is evidence that they may not prevent fading^7,8^ and might purposefully orient gaze as do macrosaccades^9^. Nonetheless, they also have undesirable properties. One is that they induce saccadic suppression^10–14^, evidenced by elevated brightness thresholds of ~ 100 ms around the time a microsaccade occurs^11,12,15–17^. Microsaccades also likely create image blur because their eye velocity is too high for sluggish retinal dynamics to compensate (> 3°/s for 0.5° saccade; see^3^). Since the retina jumps rapidly to a new position during microsaccades, they are detrimental to retinal imaging techniques such as optical coherence tomography (OCT)^1^, which require the retina to be perfectly stable. Furthermore, microsaccades may interfere with visual or oculomotor task performance, as they likely require attention^18–20^. The simultaneous attentional demands of both the microsaccades and the task create a dual task situation^21^, which may result in poorer performance of one or both behaviors.

Slow smooth movements during fixation are generally considered to be less purposeful than microsaccades, and as such they are often referred to as “drift.” While there is evidence that slow eye movements can maintain fixation accuracy without the help of microsaccades^22^, they are usually regarded as generated by noise, moving the eyes to deviant positions from which microsaccades correct^23^. More recently, a new purpose has been attributed to smooth movements, as they are thought to enhance the visibility of high spatial frequency information, and thereby improve visual perception^24^. Thus, unlike microsaccades, drifts might not interfere with task performance during fixation and could even facilitate it, though they could still contribute to retinal image instability.

Our previous smooth pursuit work showed that catch-up saccade frequency during pursuit depends on the structure of the stimulus. In seminal work, dramatically fewer catch-up saccades occurred while pursuing a 10° diameter random dot cinematogram (RDC) with coherent dot motion than while pursuing a single spot^25,26^. We suggested that fewer catch-up saccades occurred because not only did the RDCs boost pursuit by providing a better motion signal, hence mitigating the need for catch-up saccades, but that RDCs also lacked consistent foveal position error that catch-up saccades correct. A subsequent study^27^ found direct evidence that the fewer observed saccades were related to the lack of a foveal target in the RDCs, and not just their size. In that study, pursuing a peripheral target composed of four dots in a diamond configuration elicited significantly fewer catch-up saccades than either a single central spot or the 4-dot diamond with an added central spot. Since the reduction in catch-up saccades was not accompanied by changes in either pursuit latency or steady-state pursuit gain, it was concluded that the central spot elicited the catch-up saccades.

Since there is evidence that catch-up saccades during pursuit are generated by the same mechanism as microsaccades^28^, we hypothesized that using a fixation target defined by peripheral elements would result in fewer microsaccades than the standard small foveal spot, and better eye stability. In support of this, we show that fixating peripheral stimuli increases retinal stability by decreasing microsaccade rate and reducing drift speed. Furthermore, the decrease in microsaccades appears to occur primarily because the stimulus lacks a central element.

## Results

### Absence of a foveal fixation element reduces microsaccades

**Figure 1:**
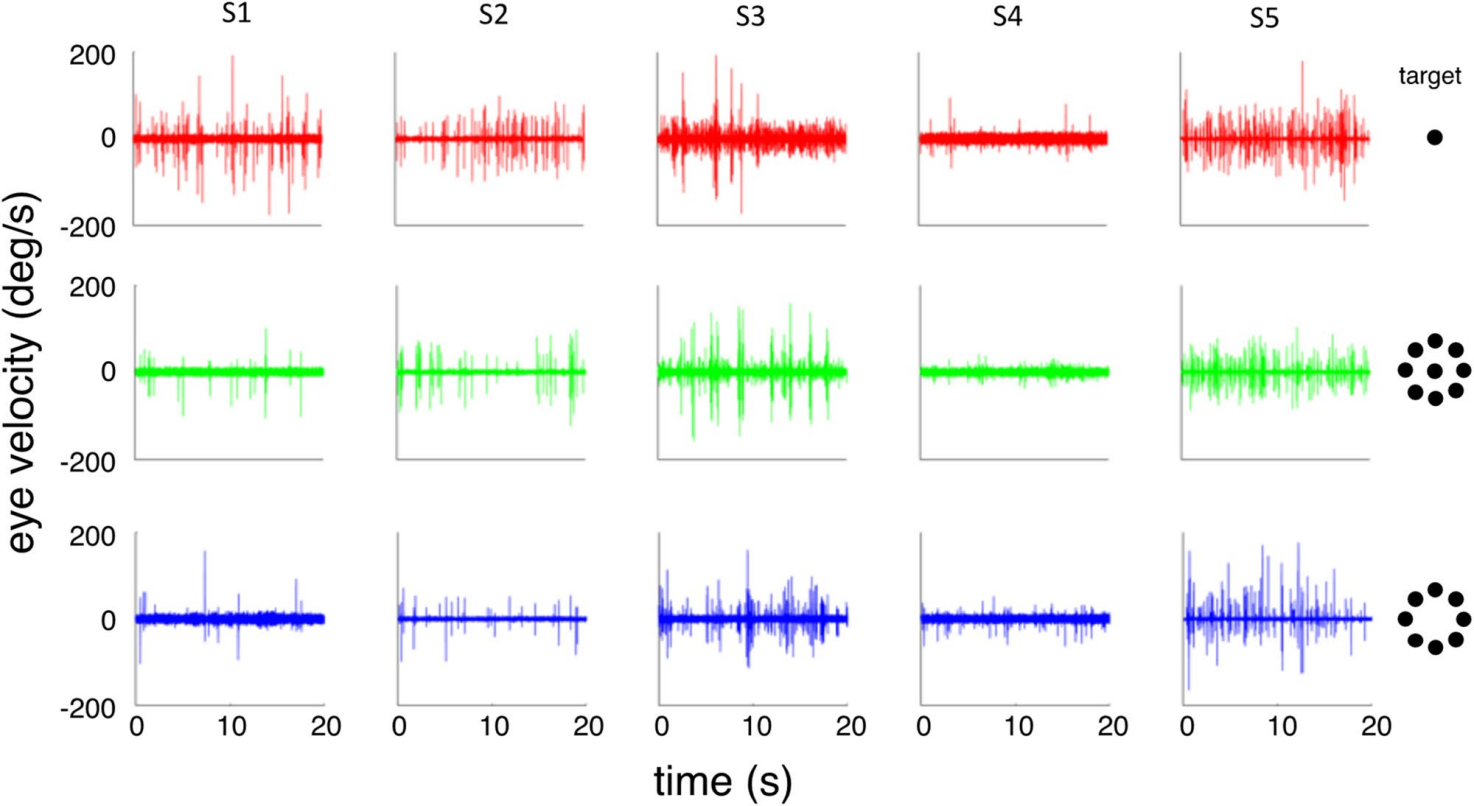
Eye velocity as a function of time for 8 randomly chosen 20 s trials for each of the five observers (columns) fixating each of the three targets (rows). Saccades appear as “spikes” in the eye velocity traces. Icons depicting the targets are at right. Note generally fewer saccades for the 8-dot peripheral target condition (bottom row).

**Figure 2:**
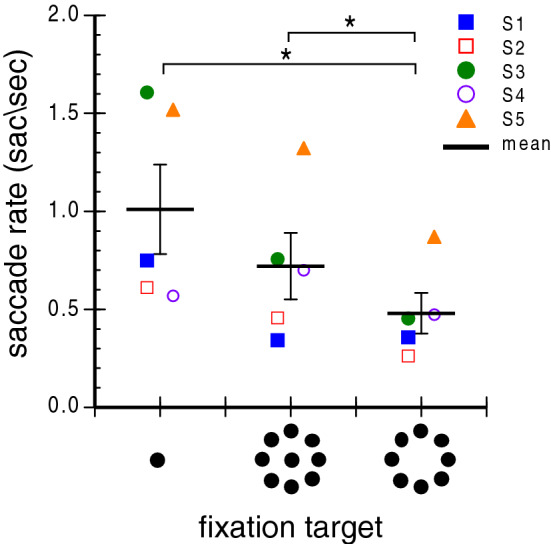
Microsaccade rate during fixation of the three targets for each observer. Mean rates are shown by the thick black lines. Error bars are ± 1 standard error. Asterisks identify significantly different conditions.

**Figure 3:**
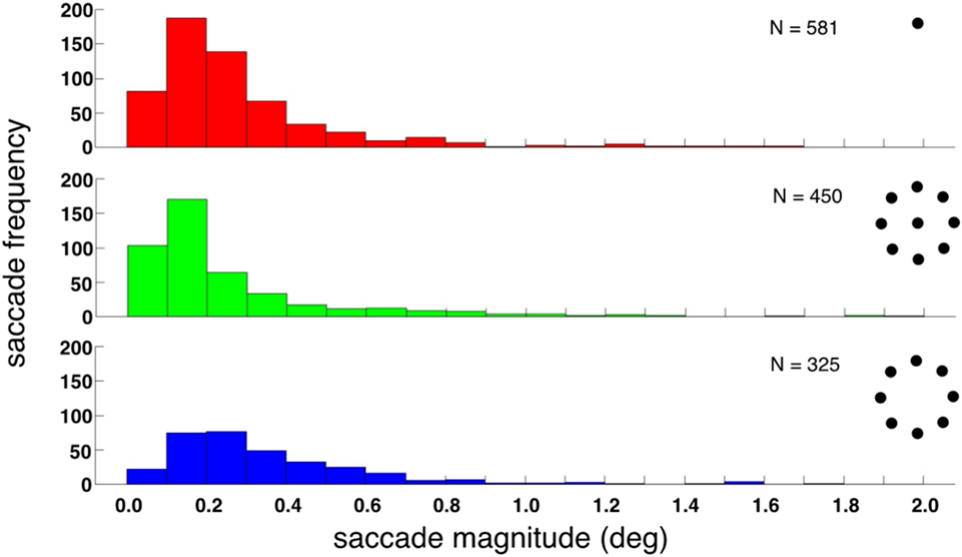
Saccade frequency as a function of saccade magnitude for the three stimulus configurations. The total number of saccades obtained for each condition appears in the top right of each panel.

Figure 1 shows eye velocity traces from eight randomly selected 20 s trials for each of the five observers fixating each of three fixation targets: a small spot, a small spot surrounded by eight identical spots in the periphery arranged in a ring, and the ring of spots alone. Each column shows data from a single observer and each row shows data for one target type. In each trace, microsaccades appear as spikes in the eye velocity trace. Note that, on average, fewer saccades occur while fixating the peripheral, 8-dot target (bottom row) than the other two targets. This effect is quantified in Fig. 2 which plots the microsaccade rate for the three different fixation targets. The mean for each condition is shown as a thick black line and individual observer data indicated by the symbols. Analogous to our pursuit result^27^, average microsaccade rate was lower for the peripheral array alone than for a single dot, but again increased when a central dot was added to the 8-dot array. Because we hypothesized fewer microsaccades, one-tailed paired t-tests compared microsaccade rate across conditions and showed that the peripheral stimulus (8-dot, 6° diameter) elicited fewer microsaccades (*M* = 0.48 sac/s) than did the central one (*M* = 1.0 sac/s) (t(4) = 2.8, *p* = 0.025) or the central + peripheral (9-dot, 6° diameter) stimulus (*M* = 0.72 sac/s) (t(4) = 3.05, *p* = 0.019). Microsaccade rate was not significantly different for the two stimuli that had a central element (1-dot vs. 9-dot; *p* = 0.086). The effect of stimulus configuration can also be seen in Fig. 3 which plots saccade frequency as a function of saccade magnitude. Across all three conditions, on average 85% of saccades were 0.5° or smaller and just over 96% of all saccades were 1.0° or smaller.

The proportion of larger saccades in the 8-dot distribution appeared to be greater than in the other two distributions. To determine if this was true, we first tested if the data were normally distributed using the Shapiro–Wilk test, and found that it was not. We therefore first converted the data to ranks before conducting a one-way repeated-measures ANOVA^29^. The ANOVA showed that differences in distributions of saccade magnitude across the three conditions just barely failed to reach significance (F(2,8) = 4.41, *p* = 0.051). The tendency of the 8-dot data to be skewed towards larger saccades may be because the central dot in the other two stimulus configurations provides a reference “anchor” for microsaccade generation, hence limiting their size.

Since elevated saccade frequency was observed with both the 1 and 9-dot stimuli, it might be that more microsaccades were being generated to correct position error between the fovea and central spot as has been shown previously to occur during single-spot fixation (e.g.^9^). To determine this, we examined whether there were more saccades directed toward the target center with the 1 and 9-dot stimuli than the 8-dot one. Saccades were classified as either directed ‘inward’ or ‘outward’ by determining their beginning and end points locations relative to center. If a saccade’s end point was closer to the center than its start point, it was classified as inward-directed. Saccades with end points farther from the center than their start points were categorized as outward-directed. Because the data were normally distributed (Shapiro–Wilk tests), a one-way repeated measures ANOVA compared the proportion of ‘inward’ microsaccades across conditions. Curiously, the proportion of ‘inward’ saccades was not significantly different between the three target conditions (1-dot: *M* = 0.63, se = 0.05; 9-dot: *M* = 0.53, se = 0.06; 8-dot: *M* = 0.57, se = 0.03) (F_2,8_ = 0.783,* p* = 0.489).

### Peripheral fixation targets reduce drift rate

Slow drifts occuring during fixation have been attributed to noise, or “fundamental instability of the oculomotor apparatus”^7^. Once drift takes the eye sufficiently far from the fixation target, a microsaccade is initiated to realign the eye with it^9^. In addition, drifts are hypothesized to aid perception of high spatial frequencies^24^ in a drift-speed dependent fashion^30^.

However, we surmised that while drifts may be initiated by noise, they are controlled by the retinal motion signals that arise after the eye begins to move. This hypothesis arises from results of our previous smooth pursuit research suggesting that catch-up saccades during fixation and microsaccades during pursuit are driven by the same mechanism^28^, and the observation that functionally, fixation and pursuit stabilize retinal images. These observations suggested to us that fixation and pursuit might be controlled by similar mechanisms. Since the main driving signal for pursuit is motion, might fixation also employ a motion signal? If so, since larger moving stimuli facilitate pursuit, presumably because they create a stronger motion signal^25^, fixating the larger stationary stimulus might produce a stronger motion signal when the eyes drift across it. This larger motion signal should be easier to detect and result in the initiation of compensatory eye movements to reduce the drift.

**Figure 4:**
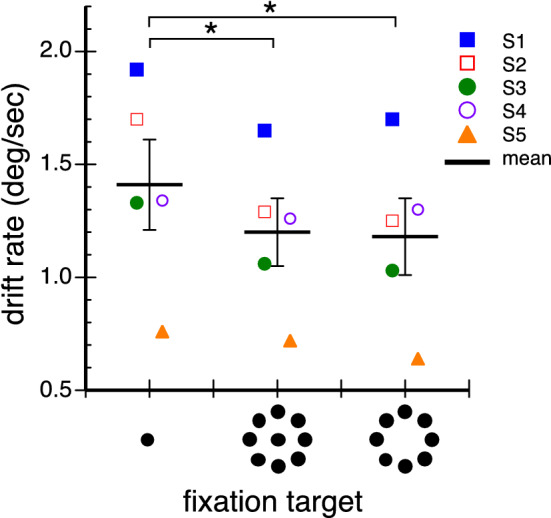
Drift speed during fixation of the three targets for each observer. Mean rates are shown by the thick black lines. Error bars are ± 1 standard error. Asterisks identify significantly different conditions.

Figure 4 plots the drift rate for the three different fixation targets. Mean drift speed for each condition is shown as a thick black line with data for individual observers indicated by the symbols. Paired one-tailed t-tests showed significantly lower drift rates in both conditions where peripheral elements were present relative to during fixation of the 1-dot central stimulus (central vs peripheral: t(4) = 3.20, *p* = 0.016; central vs central + peripheral: t(4) = 3.12, *p* = 0.018). However, the two peripheral conditions were not significantly different from each other (*p* = 0.276). Thus when peripheral elements were present, drift rate remained low even when a foveal element was also present.

### A foveal element anchors fixation

**Figure 5:**
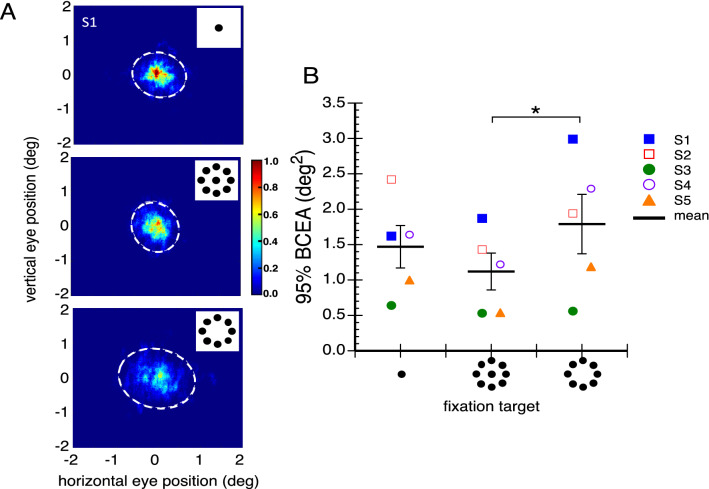
Eye position variability. (**A**) Probability of gaze locus illustrated by heat maps of eye position measured during fixation of the three targets for observer S1. Dashed circles define the 95% BCEA. (**B**) Mean 95% BCEA (deg^2^) for each of the three fixation targets for each observer. Mean overall BCEA is shown by thick black lines. Error bars are ± 1 standard error. Asterisks identify significant differences.

While peripheral stimuli may better stabilize the eyes by reducing drift speed and microsaccade rate, a foveal element might still benefit fixation by providing a better position signal to anchor it and hence maintain gaze consistency over time. Figure 5A shows probability distributions of the relative frequency of different eye positions during 20 s fixations of each of the three stimuli for one representative observer (S1), overlaid with a two-dimensional standard deviation of eye position known as a bivariate contour ellipse area (BCEA). We used the BCEA to quantify fixation variability as in previous literature^22^ (see “Methods”). Note that the peripheral array alone (Fig. 5A bottom plot) resulted in greater eye position dispersion than did the 1 and 9-dot conditions in which the central dot was present. Figure 5B shows the average 95% BCEA for each condition (thick black lines) with data for individual observers shown as symbols. Paired t-tests (two-tailed, because we did not have a clear directional hypothesis) showed a significant difference only between the two conditions with peripheral stimuli; the 8-dot peripheral stimulus’ BCEA was larger than that of the 9-dot stimulus (t(4) = 3.37, *p* = 0.028).

While the BCEA is a description of the variability of eye positions during fixation, it does not capture the spatial distribution of those eye positions, which can differ between conditions as observed in the heat maps (Fig. 5A). To better characterize eye-position distribution, we defined circular regions centered on the targets whose size was systematically increased. The smallest circle had a diameter of 0.2° and the diameters of successive circles were increased in 0.2° increments. We then computed the relative density of eye gaze samples that fell within each successively-sized circular region.

**Figure 6:**
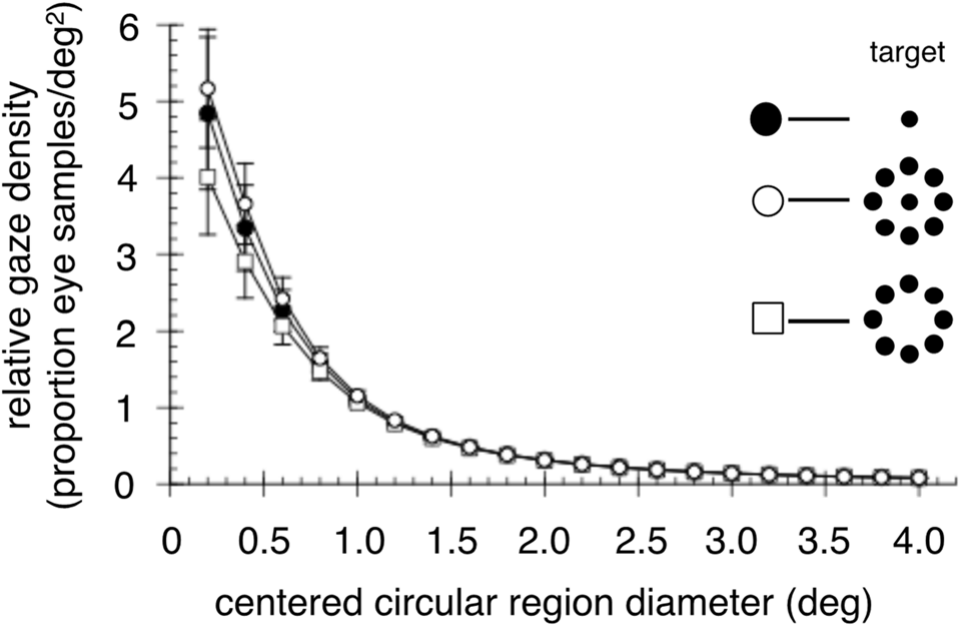
Relative density of eye gaze falling within different sized circular regions centered on the fixation targets. Error bars are ± 1 standard error. Note the highest density of eye position occurs for the central + peripheral target closest to the target center.

Figure 6 plots relative eye gaze density as a function of the diameter of the circular analysis area. Notice that while there are differences between gaze density at the smallest diameters, these differences decrease as the circular analysis region diameter increases. To test for differences in relative gaze density among the conditions, and because Shapiro–Wilk tests confirmed that the data were normally distributed, we submitted eye gaze density data for circular diameters of only 0.2°–0.8° (as this is where the largest differences occurred) to a two-way repeated measures ANOVA with target and circular region diameter as variables. The ANOVA showed that, not surprisingly, the main effect of circular region diameter was significant with smaller diameters having higher densities (F_3,12_ = 20.74, *p* = 0.0001) and the main effect of target was not significant (F_2,8_ = 2.645, *p* = 0.13). Importantly, the interaction between circular region diameter and the target was not significant (F_6,24_ = 1.75, *p* = 0.152). This shows that the relative gaze densities, and thus the spatial distribution of eye gaze during fixation, was not statistically different between the three targets even for small regions surrounding the fixation target center. The difference between the BCEAs (Fig. 5) was thus likely driven by a relatively small number of “outlier” gaze samples that fell well outside the central area.

## Discussion

Fixation is usually studied with a small central spot, and abundant literature characterizes the behavior and function of miniature eye movements while fixating it. We additionally assessed fixation with a peripheral ring of dots presented both with and without a central element. We found that these novel fixation stimuli generated substantially different fixation characteristics from those previously documented. When fixating the 8-dot peripheral stimulus, microsaccade frequency was markedly reduced (see Fig. 2), as was drift speed (see Fig. 4). Adding a central dot to the stimulus increased microsaccade frequency but drift speed remained low. Eye position variability, as assessed by BCEA, was lowest when the central and peripheral stimuli were combined (9-dot target, see Fig. 5B). However, gaze density assessed at successively larger centered circular regions showed that the 9-dot target kept the eye closer to the center than did the 1-dot or 8-dot ones (see Fig. 6), but these differences decreased as the analyzed circular region diameter increased. Nonetheless, none of the gaze density differences reached statistical significance.

Our data show that larger, peripheral fixation targets reduce microsaccades by on average 46.3%. Other work also found reduced microsaccades with different fixation stimuli. McCamy et al.^31^ found microsaccade rate decreased as the size of the fixation spot increased (up to the maximum size tested of 1°). Thaler et al.^32^ examined microsaccade rate as well as fixation variability for a variety of fixation targets ranging in size from 0.2° to 1.5° (see their Fig. 1) and concluded that a small (0.6°) conglomerate stimulus, consisting of a bullseye and crosshair, served as the ‘best’ fixation target. However, none of the targets in these previous studies exceeded 1.5° in diameter so it was unknown whether peripheral targets benefit fixation. Our results extend previous results with central targets by showing that a peripheral fixation target dramatically reduces the microsaccade rate. Importantly, it is not only the presence of the peripheral stimulus elements but also the lack of a small central one that is critical for minimizing microsaccade production.

In addition, we found that peripheral fixation targets significantly reduced drift speed, contributing to the greater fixation stability achieved when using them. Notably, the peripheral stimulus alone, on average, produced the largest fixation variability (95% BCEA, see Fig. 5B), consistent with data reported by Steinman^33^. Even so, the largest individual 95% BCEA for this stimulus was ~ 3.0 deg^2^ which means that even over a 20 s trial, the eyes remained within a circular region with a radius slightly less than 1°. Moreover, the average 95% BCEA for the peripheral stimulus was not statistically different from that obtained with the central target. The central + peripheral target produced a BCEA smaller than the central spot, but that difference was also not significant. This was confirmed with a subsequent analysis on eye position density computed over circular regions with different diameters centered on the stimuli. The central + peripheral target kept the eye closer to the target center than did the 1-dot target while the opposite was true for the solely peripheral target. However, these differences also did not reach significance.

Our results provide further evidence that microsaccades during fixation and catch-up saccades during pursuit are controlled by similar mechanisms. Microsaccades and catch-up saccades are usually considered to be different oculomotor behaviors, likely because pursuit and fixation are thought to be controlled by independent processes^34^. The independence of these systems seems reasonable given that pursuit follows moving objects, and fixation stabilizes gaze on stationary ones, yet pursuit is portrayed by the models as stabilizing retinal images, consistent with the role of fixation. Furthermore, catch-up saccades are thought to be driven by retinal position and velocity errors^35,36^, error signals that also contribute to microsaccade generation^23^. Moreover, we previously reported that the timing of microsaccade and catch-up saccade onset is controlled by a similar mechanism, since both eye movements quiesce similarly around sudden or predictable stimulus events^28^. The current results are further evidence of common microsaccade and fixation control since they suggest that microsaccades are elicited by foveal stimuli, as our previous finding suggests also occurs with catch-up saccades during pursuit^27^.

Our study has implications for understanding the function and control of microsaccades. Their role in vision has been, and still is, controversial (for a review see^3^). It was initially thought that they were generated by a stochastic process, and that they prevent retinal images from fading when the eyes are completely stationary^37^. However, retinal fading occurs with or without microsaccades, and hence, reducing fading may not be their purpose^7^, though there is ardent support that they mitigate fading to a degree^5,6^. More recent work suggests that microsaccades are not generated by noise but are instead controlled. As such, they correct for position error between the target and the eyes and can also indicate the perceptual/behavioral goal^9^. Our current results also argue against a passive role of microsaccades in reducing image fading, as microsaccade rate varied with the structure of the fixation target. Furthermore, the eyes drifted more slowly with the larger peripheral stimulus than with the single foveal dot. If microsaccades merely refresh a retinal image, fewer of them should be required with a single dot given the faster drift.

The current results are the first to elucidate a mechanism underlying drift control during fixation. While evidence exists that drift is controlled in order to keep the eyes near a fixation spot^22,38^, it is generally thought to be generated by a noise process (e.g.^23^). However, in our study less drift accompanied larger stimuli. We deduced that this was because the larger stimuli created a stronger motion signal in the visual system when their image drifted across the retina. This deduction was fueled by our analogous results with the pursuit system showing that larger stimuli reduced catch-up saccades but increased open-loop acceleration, likely because larger stimuli produce a better motion signal^27^. Therefore, smooth eye movements during fixation might be controlled by a feedback system that uses motion signals produced when the eyes drift across a stationary target in order to stabilize the eyes. This mechanism is analogous to, and possibly the same as, the feedback mechanism the pursuit system uses to minimize the retinal-image velocity of a moving target^39,40^.

Our results could improve the clinical assessment of the retina. In perimetry tests for retinal dysfunction (e.g., macular degeneration), a patient fixates a central spot while they detect small probes flashed briefly at locations spanning the retina (e.g.,^41^). If the flash occurs during a microsaccade, its detection could be impaired because of saccadic suppression, excessive retinal motion, or even because fewer attentional resources are available to detect it. Stimuli exist for maintaining fixation without a central dot when there is damage to the central retina. These stimuli are constructed using radial gratings that are wide in the periphery and taper narrower as they approach the center (e.g.,^42–44^). However, they apparently afford no benefit to gaze stabilization as Gonzalez et al.^43^ found no significant difference in the 63.2% BCEAs during fixation of a central spot (0.5°) or a 5° radial grating centered at the fovea. Radial grating targets may also minimize microsaccades, but data on this is lacking. Using a spot fixation target also limits the efficiency of in-vivo imaging obtained using optical coherence tomography (OCT). In OCT, retinal “slices” are obtained by scanning the retinal tissue at various depths and collecting reflected light from retinal tissue. Microsaccades interrupt the scanning process, and frames where microsaccades occur must be deleted^1^. Our work provides a method by which microsaccades are minimized, hence preserving scanning frames in OCT leading to more accurate and efficient retinal imaging.

Our results provide evidence that despite small foveal targets being employed to maintain fixation in most vision-science experiments, they may not be the best choice. If the purpose of a fixation target is to keep the eyes centered, a central + peripheral target appears optimal. Furthermore, such targets appear to decrease slow drifts that are common with small single-dot targets. However, if the goal of fixation is to maximize ocular stability, a peripheral target alone appears to be the most effective, as it reduces microsaccades as well as drift speed.

## Methods

### Participants

Five healthy experienced humans were observers in the experiment (four males and one female). Two of them are authors, and three were naïve to the experimental aims. All had normal or corrected to normal vision and were 25–54 years old. Experimental protocols were approved by the Smith-Kettlewell Institutional Review Board and the Wright State University Institutional Review Board, and all experiments were conducted in accordance with the Declaration of Helsinki. Observers gave informed consent prior to participating. Two observers’ data (S3, S4) were collected at Wright State University (Dayton, OH) and three observers’ data (S1, S2, S5) were collected at the Smith-Kettlewell Eye Research Institute (San Francisco, CA). Collecting data at two locations increases the reproducibility and generalizability of the results.

### Apparatus

Visual stimuli were generated in MATLAB (MathWorks, Inc., Natick, MA) using the PsychToolbox^45,46^. At Smith-Kettlewell, experiments were run on a Macintosh Macbook Pro laptop with stimuli presented on a 17-inch, high resolution Nanao color monitor (1.76 min arc/pixel) at a rate of 60 Hz. At Wright State, experiments were run on a Macintosh Pro computer with stimuli presented on a 23-inch, high resolution Samsung color monitor (1.57 min arc/pixel) at a rate of 120 Hz. At both locations, an EyeLink 1000 video-based eye tracker (SR Research Ltd., Mississauga, Canada) recorded monocular horizontal and vertical eye positions at 1000 Hz. The eye trackers were calibrated and validated using the EyeLink’s standard nine-point method. A chin and forehead rest stabilized the observer’s head and maintained a constant viewing distance of 48 cm (CA) or 57 cm (OH). At both locations, monitors were set to their maximum luminance and brightness levels, and stimuli were presented at the same grey-scale values (R:142, G:142, B:142). The same experimental programs were run at both locations. We did not equalize monitor luminance across locations as stimuli were far above threshold values and as such, small deviations in luminance would have little impact on performance.

### Stimuli and procedure

**Figure 7:**
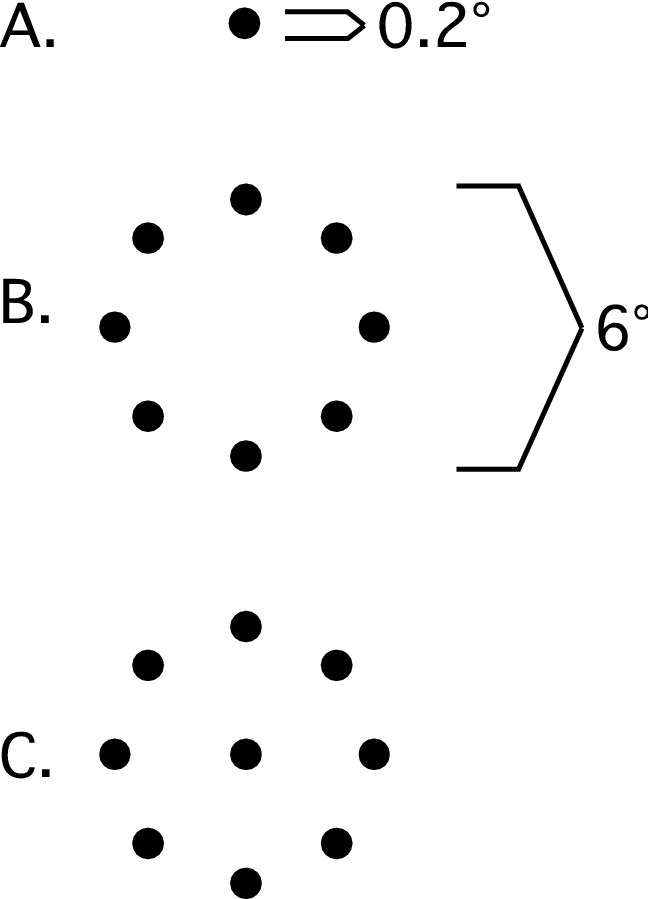
Schematic illustration of the three fixation targets.

In separate blocks, observers fixated one of three fixation target configurations (luminance 2.6 cd/m^2^) presented on a dark background (luminance 0.3 cd/m^2^) (see Fig. 7). One was a single small gray dot (0.2 deg diameter). The second comprised eight identical gray peripheral dots (0.2 deg diam.) equally spaced to form a virtual circle (6.0° diameter). The third target was a composite stimulus of both the central and peripheral dots.

Each trial was initiated by a keypress, following which the fixation target appeared. Observers then fixated the target for 20 s. Following each 20 s trial, the fixation target disappeared, and the observer initiated the next trial when ready. Fixation conditions were blocked. For all three conditions, a minimum of eight 20 s trials were collected from each observer. Block order was randomized. Each block of eight trials took approximately four minutes to complete. The three naïve observers were given at least 5 trials of practice before experimental data were collected.

### Eye movement analysis

Horizontal and vertical eye velocities were calculated offline from the recorded eye position signals by differentiating and filtering the raw position data (2-pole Butterworth non-causal filter, cutoff = 50 Hz). Saccades were detected using the Eyelink’s saccade detection routine utilizing 0.1 deg displacement, 30 deg/s velocity, and 8000 deg/s2 acceleration thresholds. Using custom MATLAB code, we analyzed and visualized each eye movement trial. Saccades were confirmed by visual inspection of all eye velocity and position traces by trained researchers. Any saccades that occurred near blinks were excluded as were any erroneously detected saccades, either due to blinks or noise (181 saccades excluded, 11.67%). Post-hoc analysis was done on microsaccades only, by excluding saccades with magnitudes greater than 2 deg consistent with previous studies^5,32,47^. This procedure resulted in the exclusion of an additional 14 (0.9%) saccades. Drift rate was quantified by calculating the instantaneous radial speed between successive pairs of position samples and then averaged over the entire trial after removing saccades and blinks. Bivariate Contour Ellipse Area (BCEA)^33^ was calculated with a 95 percent confidence interval using the following equation:

1 $$\left( {\frac{x}{{\sigma_{x} }}} \right)^{2} + \left( {\frac{y}{{\sigma_{y} }}} \right)^{2} = 5.991$$

where *x* and *y* represent the horizontal and vertical coordinates for each point in the data set, and ${\sigma }_{x}$ and ${\sigma }_{y}$ represent the standard deviation of the x and y components. The value 5.991 is the scale of the ellipse and corresponds to the two-degree of freedom chi-square distribution value for the 95 percent confidence interval. The ellipse was then transformed by determining the eigenvectors based on the covariance of the data and rotating the data by the angle between the largest eigenvector and the x axis so that it is aligned with that eigenvector. The BCEA plot was overlaid on top of a heat map obtained by creating a 2-D contour plot using the frequency distribution of the x and y coordinates of the data along with the 2-D grid coordinates for those points^23^.

## Data Availability

The datasets used and/or analyzed during the current study are available from the corresponding author on reasonable request.
